# Personality traits, role ambiguity, and relational competence as predictors for teacher subjective wellbeing

**DOI:** 10.3389/fpsyg.2022.1106892

**Published:** 2023-01-05

**Authors:** Crenguța Mihaela Macovei, Ștefania Bumbuc, Fabiana Martinescu-Bădălan

**Affiliations:** Department of Applied Social Sciences and Humanities, “Nicolae Bălcescu” Land Forces Academy, Sibiu, Romania

**Keywords:** teacher subjective wellbeing, personality, role ambiguity, relational competence, university teachers

## Abstract

The coronavirus pandemic has significantly reshaped the way teaching activities are carried out, thus intensifying the stress felt by teachers. The teacher-student relationship has also changed under the influence of social constraints. Together, these have affected teachers’ work efficiency and redefined their connection with the school. The present study aims to examine the extent to which personality traits, role ambiguity, and relational competence predict teacher subjective wellbeing. The study sample consisted of 105 university teachers. Three hierarchical multiple regression analyses were conducted separately for each of the three criterion variables used in this research: *teaching efficacy*, *school connectedness,* and *teacher subjective wellbeing*. The results indicated that the personality traits *emotionality*, *extraversion*, and *conscientiousness* are significant predictors for all three variables, while *honesty-humility*, *agreeableness,* and *openness to experience* are not predictors for any of the variables. However, in the third step of the regression analysis, *conscientiousness* was found to lose its predictive quality for the variables *school connectedness* and *teacher subjective wellbeing*, its place being taken by *emotionality*. Both *role ambiguity* and *relational competence* are significant predictors for *teaching efficacy*, for *school connectedness,* and for *teacher subjective wellbeing*. Based on these results, universities can design some measures to reduce role ambiguity of teachers and can identify areas of training needed to increase their relational competence, while simultaneously reducing the costs associated with wellbeing and productivity problems. Several training modules and courses are proposed to be designed and included in the curriculum of initial and in-service teacher training programs, in order to contribute to increasing teachers’ performance.

## 1. Introduction

In order to manage technological progress and social change, people need to constantly develop new skills—they need high-quality education. Therefore, educators are constantly being given new and increasingly complex tasks and a particular social responsibility to prepare children, young people, and adults for the demands of today’s world and for the future. There is a consensus in the literature that the teaching profession is particularly challenging and demanding, that social responsibility is at a high level, and that teachers are expected to perform at their best, with dedication, availability, and commitment (Kebritchi et al., 2017; la Velle, 2020). Compared to many other professions, the teaching profession also has a strong vocational component (Köpsén, 2014), as it is not just about applying teaching techniques or methods of working with students (Balaş and Bran, 2014), but is also done “with the soul,” involving the personality traits of the teacher to a large extent.

In recent years, however, it can be observed that the teaching profession is becoming less and less attractive (European Education and Culture Executive Agency et al., 2019; European Commission et al., 2021), and the level of motivation and job satisfaction is decreasing among teachers (Skaalvik and Skaalvik, 2011). As in other fields of work, in the post-COVID-19 pandemic period, there is also talk about the phenomenon referred to in the press as “The Great Resignation,” even in the field of education. The level of professional stress is high among teachers, pressure, and burnout being among the most important reasons for leaving the profession (O’Brennan et al., 2017). We are currently experiencing a marked reduction in interest, motivation, and professional commitment, while an unexpectedly large number of teachers are increasingly willing to change their job or even their profession (Steiner and Woo, 2021; Nguyen et al., 2022). In the context of the need to understand the factors and reasons that determine these types of attitudes and decisions of teachers, great emphasis must be placed on the teachers’ self-reported levels of wellbeing, their personality traits, the ways they relate to each other in school and the roles they are expected to fulfil.

There are three main research orientations related to the concept of wellbeing: social wellbeing, psychological wellbeing, and subjective wellbeing (Huang et al., 2022). In what regards the objective approach of the term, Kahneman (1999) defines wellbeing as “objective happiness” referring to the quality of life measured in terms of health, material status, access to education or social services, etc.

Bradburn (1969) states that psychological wellbeing occurs as a consequence of the dominance of positive affects over negative effects (high wellbeing) or vice versa (low wellbeing).

The term “subjective wellbeing” refers to people’s emotional and cognitive evaluations of their lives (Diener and Lucas, 1999; Alexandrova, 2005); in this case, people use personal standards to make general judgments about the quality of their lives at a given point in time and are expressed by what they call happiness, peace, fulfillment, and life satisfaction (Diener et al., 2003). In this paper, the focus is on teacher subjective wellbeing, i.e., the theoretical construct around which the research hypotheses will be formulated, and the empirical research will be structured.

## 2. Teacher subjective wellbeing

Research on subjective wellbeing has emerged as an important area of focus for psychology in this millennium (Pavot, 2008). The subjective wellbeing typically refers to the self-reports on health (physical, emotional, and spiritual), success, work-life balance, and to the result of the individual’s evaluation of his/her own life, so that he/she may (or may not) consider it a fulfilled existence (Diener and Seligman, 2004). Subjective wellbeing is the result of an overall assessment of the quality of life from the person’s own perspective. It is a perception of the extent to which “a person believes or feels that his or her life is going well” (Diener et al., 2018, p. 15).

“Because subjective wellbeing refers to affective experiences and cognitive judgments, self-report measures of subjective wellbeing are indispensable” (Eid and Larsen, 2008, p.4). The indicators used to measure subjective wellbeing include emotional components, based on the balance between positive affect and negative affect, and cognitive components, based on life satisfaction. The subjective wellbeing has been explained in a hedonic approach as the way a person uses everything around him/her in order to gain utility and develop positive emotions, based on external conditions and constructive experiences (Bradburn, 1969; Kahneman, 1999).

In what regards the teachers’ work lives, some of the studies on teacher subjective wellbeing approach the issue from the perspective of experiencing positive emotions and a high level of life satisfaction, describing subjective wellbeing as teachers’ positive psychological functioning at work (Renshaw et al., 2015a). Other, more numerous studies (*cf.* Renshaw et al., 2015a), analyze subjective wellbeing from the perspective of a low level of negative mood or affect, looking at indicators such as occupational stress and coping mechanisms (Lambert et al., 2009; Parker et al., 2012) or burnout (Aluja et al., 2005; Maslach and Leiter, 2008; Fleming et al., 2013). Teachers coping strategies can predict occupational wellbeing (Parker et al., 2012). There are also combined approaches in which subjective wellbeing is described as the result of the ongoing balance between positive and negative aspects of working life, such as between levels of engagement and burnout (Parker et al., 2012) or between teacher efficacy and burnout (Pas et al., 2012).

Teacher wellbeing is “an individual sense of personal professional fulfillment, satisfaction, purposefulness, and happiness, constructed in a collaborative process with colleagues and students” (Acton and Glasgow, 2015, p.101).

As the sum (effect) of positive aspects of a teacher’s professional life, subjective wellbeing is analyzed and measured in terms of various indicators, such as teaching efficacy and experiencing positive emotions at work (van Horn et al., 2004), resilience level (Beltman et al., 2011), manifesting pro-social attitudes and relations (Hascher and Waber, 2021), etc. The theoretical multidimensional model of occupational wellbeing of teachers proposed by van Horn et al. (2004) includes five dimensions: affective, cognitive, professional, social, and psychosomatic. The overall level of wellbeing results from combining the intensity of positive or negative manifestations of these dimensions. Positive emotions have emerged as the fundamental predictor of overall subjective wellbeing (van Horn et al., 2004).

The importance of using positive indicators of teacher subjective wellbeing, such as positive emotions and cognition, has been highlighted in studies that have related teacher wellbeing to student wellbeing (Renshaw et al., 2015b), students’ motivation and teaching efficacy (Nie et al., 2012). The quality of the relationship with students as well as the ability of teachers to support their students in times of crisis also condition the level of teacher wellbeing (Falk et al., 2022).

As a result of empirical research, it has been found that there is no significant inverse correlation between positive and negative experiences involved in subjective wellbeing. Attempting to reduce teachers’ negative states does not necessarily result in an increase in their positive states, and an increase in subjective wellbeing does not automatically occur when there are no threats or barriers to wellbeing (Diener et al., 2005). Objective circumstances may not explain all the variance in wellbeing judgments (Diener et al., 2018). In the absence of this relationship, in order to understand how subjective wellbeing manifests itself, it proves equally necessary and useful to discuss inter-individual differences and the role that teacher’s personality may play in their subjective value judgments about their own lives. Thus, it is possible that some of the cognitive and affective components of subjective wellbeing may be predicted by certain personality traits, by the level and sources of teachers’ stress, and by the quality of the relationships with others.

## 3. School connectedness

School connectedness is a particular form of social connectedness, which teachers experience at the workplace. Social connectedness refers to the experience of belonging to a social relationship or network and includes a person’s subjective awareness of being in close relationship with the people around. Social connectedness is a mediator in the relationship between extraversion and subjective wellbeing (Lee et al., 2008). Having supportive relationships is one of the strongest predictors of wellbeing, with a notably positive effect (Myers, 2003).

School connectedness manifests as a result of the interaction between two inter-dependent emotions and attitudes (Monahan et al., 2010, p. 3): “school attachment (that entails close affective relationships with school community) and school commitment (characterized by an emotional investment and the sensation of doing well in school).”

Creating a sense of connectedness among staff members establishes a positive organizational climate in the school, and, as a result, student and teacher involvement in all activities increases, and student or teacher dropout is greatly reduced (Thapa et al., 2013). Furthermore, teachers who feel a sense of belonging in their school communities have a strong connection with their students and with the school management and tend to feel less professional burnout (O’Brennan et al., 2017). For teachers in their first years of activity, close interpersonal relationships and the support received from colleagues and administrative staff is very important. The lack of this support from the school community is one of the reasons why teachers leave their jobs (Marlow et al., 1997). There are studies indicating that teachers’ level of job dissatisfaction and their intention to resign is proportional to the extent to which they feel less connected to their students (Martin et al., 2012).

Studies on teachers’ sense of belonging to the school are much fewer in number compared to the plethora of studies investigating the students’ sense of belonging and their connectedness to the school, as a prerequisite for good educational outcomes (Rogerson, 2004). There are surprisingly few studies investigating the relationship between the sense of belonging (or the school connectedness) of teachers and various aspects of a teacher’s work, such as teaching efficacy, job satisfaction, work motivation, and contentment, etc.

There are also no significant studies investigating the relationship between the teachers’ personality traits and the intensity of the sense of connectedness they establish with the school or the intensity of their sense of belonging to the school community. Further research on aspects of school connectedness and teaching efficacy found that teachers who feel happy at school, who feel valued and appreciated, tend to have a positive attitude toward work and, consequently, higher productivity (Battistich et al., 1997).

## 4. Teaching efficacy

Teacher sense of efficacy was described as “the belief that the teacher can help even the most difficult and unmotivated students” (Berman et al., 1977, p.136) and the extent to which a teacher believes he or she has the capacity to affect student performance (Khan et al., 2015).

Starting from the premise that the level of teaching efficacy is given by the students’ results, the teachers’ sense of efficacy is studied based on three main tasks that a teacher performs in a school environment, namely: efficacy of the instructional strategies, efficacy of classroom management, and efficacy of engagement in students’ motivation (Nie et al., 2012). During the pandemic, the scores of teacher sense of efficacy (for both instruction and engagement) were lower than in the studies conducted before the pandemic. The data also show that “teachers who are teaching virtually had the lowest efficacy scores compared to teachers teaching in a hybrid or all in-person model” (Pressley and Ha, 2021).

Using the Teacher Efficacy Scale to measure teachers’ attitude toward working with students, some studies highlighted another two dimensions of teacher efficacy: general teaching efficacy and personal teaching efficacy (Saklofske et al., 1988; Hoy and Woolfolk, 1993).

There are two approaches to teaching efficacy in the literature: (a) efficacy in relation to the students, understood as a manifestation of the teachers’ confidence that they can control or influence students’ learning activity; and (b) efficacy in relation to oneself, understood as part of self-efficacy, the cognitive process by which teachers build the confidence that they are capable of achieving a certain level of performance in their activities.

## 5. Personality traits

The teacher’s personality is one of the most important and complex variables in the educational process.

The Five-Factor Model is currently the dominant paradigm in personality research and one of the most common starting points in researching teachers’ personalities influence on the teaching-learning process (Gongz, 2017). The Five-Factor Model of personality includes the following dimensions: neuroticism, extraversion, agreeableness, conscientiousness, and openness to experience. Neurotic people are nervous, high-strung, insecure, self-pitying, impatient, jealous and envious, subjective and impulse-ridden, and emotional and impatient. Extraverted people are sociable, fun-loving, affectionate, friendly, spontaneous, talkative, active, warm and passionate, dominant, and bold. Openness describes imaginative, original, creative, curious, daring, analytical, independent, liberal, artistic people. Agreeable people are empathetic, kind, altruistic, warm and compassionate, lenient, open-minded, cheerful, gullible, straightforward, and humble. Conscientious people appreciate productivity and efficiency, and are dependable, well organized, scrupulous, self-disciplined, practical, persistent, intelligent, responsible, and ambitious (Costa and McCrae, 1992).

It cannot be argued that there are specific personality traits that predispose a person to high levels of wellbeing (McCallum, 2021), but it has been shown that the main factors that are strongly associated with wellbeing include optimism, extraversion, and self-esteem (Costa and McCrae, 1980; Diener et al., 2003; Gale et al., 2013; van Allen et al., 2021).

There are authors who recommend the use of new models or theories of personality in the study of teacher personality, such as the HEXACO model (Ashton and Lee, 2007; Gongz, 2017; McAbee et al., 2019). This model was developed by Ashton and Lee using methods similar to the Five-Factor Model but describes six personality dimensions, namely honesty-humility (H), emotionality (E), extraversion (X), agreeableness (A), conscientiousness (C), and openness to experience (O). The description of the extraversion, conscientiousness, and openness to experience dimensions from HEXACO is similar to those from the Big Five, but the individual characteristics described by the agreeableness and neuroticism from the Big-Five model are split between honesty-humility, agreeableness, and emotionality scales in the HEXACO model. Emotionality, for example, overlaps only partially with the neuroticism factor in the Big Five, because it includes both negative emotions, such as fear and anxiety, as well as emotionally neutral manifestations, such as sentimentality and dependence. In HEXACO, agreeableness refers to people who forgive easily, judge others leniently, are willing to compromise and cooperate with others, and control their anger easily. The sixth factor, called honesty-humility, is specific to the HEXACO model and correlates very strongly with the agreeableness factor in the Big-Five model. People with very high scores on honesty-humility are not interested in manipulating others for their own gain, are not tempted to break the rules, are not interested in luxury or opulence, and are not interested in elevating their own social status. People with very low scores on this factor flatter others to get what they want, pursue material gain, have an intense sense of self-importance, and are not shy about breaking rules for their own benefit (Lee and Ashton, 2013; Ashton et al., 2014). Low levels of honesty-humility correspond to high levels of psychopathy, machiavellianism, and narcissism, which are Dark Triad personality constructs; by introducing this factor into their model, the HEXACO authors filled the gap left by other measurements of the Big-Five personality traits (Paulhus and Williams, 2002; Gaughan et al., 2012; Howard and van Zandt, 2020).

“The HEXACO model can be successfully used in research when behaviors and traits found on the Honesty–Humility, Agreeableness and Emotionality dimensions are of specific interest, including the study of teacher personality. An individual teacher who scores low on the H factor may have a proclivity for anti-social acts. It is also probable that a teacher with a personality pattern of high levels of H and A, and low level of E, would have a tendency for pro-social altruistic behaviors and inclined toward forgiveness and tolerance, which is an important propensity in every aspect of the educational process.” (Göncz, 2017, pp. 90–91).

Starting from these postulations, we have formulated the first hypothesis of our research:

*H1*: Personality traits positively relate to teaching efficacy, school connectedness and teacher subjective wellbeing.

## 6. Role ambiguity

A role is a set of expectations about the desirable behaviors of a person occupying a certain position within a social structure. When these expectations are imprecise or unclear to the person, role ambiguity will manifest as the uncertainty about whether the role will be fulfilled in accordance with the expectations of others (Carter and Harper, 2016).

Alongside role conflict, role ambiguity is a dimension of role stress (Rizzo et al., 1970; Karatepe et al., 2006; Coelho et al., 2011; Schmidt et al., 2014). Some authors (Kahn et al., 1964; Peiró et al., 2001) introduce a new dimension of role stress, namely “role overload,” while others propose the term “job ambiguity” together with a tool to measure its three facets: work method, scheduling, and performance criteria (Breaugh and Colihan, 1994).

“Role ambiguity is referred to as the lack of clarity about duties, objectives and responsibilities needed to fulfil one’s role and is often experienced in technology, social and job changes.” (Urien et al., 2017, p.139). Role ambiguity is a stressor for both the role occupant and for those around them (Doherty and Hoye, 2011). The intensity of stress felt by the individual when experiencing role ambiguity is determined by their role identity salience and the degree of uncertainty felt when having to align their goals with the role. In organizations, role ambiguity is most often manifested when communication and coordination of activities are poor or when the role occupant is expected to solve tasks outside those included in the job description (Carter and Harper, 2016; Urien et al., 2017).

By analyzing the differences between the manner in which the employee describes his/her role and the way in which the organization presents it in the published job description, under the section “abilities, interests, knowledge, skills, work activities, work context, work styles, and work values,” Saha et al. (2019, p.137) observe that role ambiguity “is associated with depleted wellbeing, such as increased heart rate, increased arousal, decreased sleep, and higher stress, …with lower job performance such as decreased organizational citizenship behavior and decreased individual task performance.”

Lack of clarity about the fundamental requirements of the job can be the cause of repeated failures that consistently diminish the employee’s sense of occupational self-efficacy. Ambiguous role circumstances can deter him/her from putting even more effort into the job, effort that can create frustration, anxiety, and fatigue over time (Acker, 2003; Tomas, 2021). Role ambiguity negatively affects job performance and can result in job burnout (McCormack and Cotter, 2013; Olivares-Faúndez et al., 2014; Wu et al., 2019). Role ambiguity can also be associated with mental health problems such as depression and anxiety (Schmidt et al., 2014).

Considering the results of these studies, we have formulated the second hypothesis of our research:

*H2*: Role ambiguity negatively relates to teaching efficacy, school connectedness and teacher subjective wellbeing.

## 7. Relational competence

Relational competence (also called interpersonal competence) is a manifestation of social and emotional competence (SEC). SEC has been defined as “the ability to understand, manage, and express the social and emotional aspects of one’s life in ways that enable the successful management of life tasks such as learning, forming relationships, solving everyday problems, and adapting to the complex demands of growth and development” (Elias et al., 1997, p. 2). Teachers’ social and emotional skills are essential for developing the same types of skills in their students, but also for developing the students’ ability to learn in general (Jennings and Greenberg, 2009; Schonert-Reichl et al., 2015).

According to Jensen et al. (2015), (as cited in Aspelin and Jonsson, 2019, p. 265), “the true core of relational competence… consists of being able to meet students and parents with openness and respect, to show empathy and to be able to take responsibility for one’s own part of the relationship as an educator.”

Vidmar and Kerman extracted the specific components of this competence starting from a concept proposed by Juul and Jensen (2010) (as cited in Vidmar and Kerman, 2016). The analyzed components are:

- the ability *to see* the students, in other words, treating them as autonomous persons capable of actively building and maintaining a relationship by understanding what lies beyond their obvious behaviors;
- leadership: refers to the teacher’s ability to manage the educational process, being fully aware of the student’s individuality and taking care of his/her integrity;
- authenticity: the teacher’s ability and willingness to include personal elements in the relationship (e.g., thoughts, values, and beliefs) and to develop subject-subject relationships rather than subject-object relationships, in which the student is merely a recipient of the knowledge transferred by the teacher;
- accountability: the teacher’s ability to initiate and maintain contact with students and to develop a positive, supportive relationship with them.

The first two components of this model were grouped into one component called *respect for individuality*. In their model, Vidmar and Kerman (2016) included three components of relational competence: *respect for individuality, responsibility,* and *authenticity*. In the scale they developed, the authenticity factor was not validated; therefore, this scale measures only the first two factors.

The study of the teachers’ relational competence has largely focused on its effects on student wellbeing and learning or teacher job stress or job performance; few studies examine the link between this competence and teacher wellbeing (Friedman, 2000; Kyriacou, 2001). Personal relationships with students provide teachers with intrinsic rewards and add meaning to their work; when these relationships are conflictual, disrespectful, or distant, they experience negative affects, their efficacy beliefs deteriorate, and they begin to feel helpless (Spilt et al., 2011). In Shann (1998) study, the teacher-student relationships had the strongest impact on teacher job satisfaction. In Yoon (2002) study, negative affect, teacher stress, and negative relationships were strongly correlated, and teacher stress predicted negative teacher-student relationships.

Based on these results, we have devised the third hypothesis that underlies our research:

*H3*: Teacher relational competence positively relates to teaching efficacy, school connectedness and teacher subjective wellbeing, after controlling the effect of personality traits and role ambiguity.

## 8. Materials and methods

### 8.1. Participants

The convenience sample consisted of 105 Romanian university teachers, with academic titles of assistant professor, associate professor, and university professor. Of these, 49.5% are men and 50.5% are women, 33.3% are professors in civilian universities (“Lucian Blaga” University of Sibiu and “Aurel Vlaicu” University of Arad) and 66.7% are professors in military academies (“Nicolae Bălcescu” Land Forces Academy of Sibiu; “Henri Coandă” Air Force Academy of Brașov, and “Mircea cel Bătrân” Naval Academy of Constanța).

### 8.2. Procedure

Participation in this study was voluntary. Initially, 250 teachers were invited to take part in the research; of these, only 105 agreed to participate. The research team collected the work email addresses of the teachers in the targeted civilian universities and military academies. The owners of the email addresses received a message containing a link allowing them to access the questionnaires in electronic format, together with a brief description of the purpose of the research and measures used. Data were collected between July and September 2022, using a convenience sampling method. The questionnaire was set up so as not to collect respondents’ email addresses, thus ensuring the anonymity of the responses. The teachers were not remunerated for their participation and were informed that the research was for scientific purposes only.

### 8.3. Measures

1. *Teacher Subjective Wellbeing Questionnaire* (Renshaw et al., 2015a) is a scale consisting of eight items, assessing the teachers’ work-related wellbeing. It consists of two subscales—*teaching efficacy* and *school connectedness*; the authors indicate that scores on these two subscales can be used as stand-alone wellbeing measures, along with a general *teacher subjective wellbeing* score. The participants need to give their answers on a four-point Likert scale, from (1) almost never to (4) almost always. Cronbach’s alpha for the entire scale was 0.90, for *teaching efficacy* subscale was 0.85, and for *school connectedness* subscale was 0.87.
2. *Personality traits* were measured using *HEXACO-60* personality inventory (Ashton and Lee, 2009), a 60-item measure using a five-point Likert scale, from (1) strongly disagree to (5) strongly agree. This inventory consists of six scales: honesty-humility (H), emotionality (E), extraversion (X), agreeableness (A), conscientiousness (C), and openness to experience (O). Cronbach’s alpha for the entire scale was 0.73, and for the subscales ranged from 0.58 to 0.82 (Table 1).
3. *Role ambiguity* was measured using the five-item subscale from *Teacher Stress Inventory* developed by Schutz and Long (1988). This subscale measures the stress level of teachers as a consequence of insecurity induced by the ambiguity of their role at work. The participants were asked to give their answers on a five-point Likert scale, from (1) strongly disagree to (5) strongly agree. As recommended, positively phrased items were reversely scored in order to accurately reflect the stress level of the respondents. Cronbach’s alpha for the entire scale was 0.82.
4. *Teacher’s Relational Competence Scale* (Vidmar and Kerman, 2016) is an 11-item measurement tool that also uses a five-point Likert scale from (1) very rarely or never to (5) always or very often. The authors identified two dimensions of the teacher’s relational competence—*individuality* and *responsibility*. In our study, only the total score obtained by the respondents on the entire scale was used. Cronbach’s alpha for the entire scale was 0.83.

**Table 1:** Correlation matrix between research variables.

	Variables	*M*	*SD*	1	2	3	4	5	6	7	8	9	10	11
1	Teaching efficacy	3.36	0.60	(0.85)										
2	School connectedness	3.12	0.71	0.719^**^	(0.87)									
3	Teacher subjective wellbeing	3.24	0.61	0.914^**^	0.939^**^	(0.90)								
4	Honesty-humility	3.88	0.50	0.115	0.017	0.067	(0.72)							
5	Emotionality	2.83	0.57	−0.237^*^	−0.342^**^	−0.316^**^	0.008	(0.79)						
6	Extraversion	3.58	0.53	0.461^**^	0.518^**^	0.530^**^	0.004	−0.454^**^	(0.82)					
7	Agreeableness	3.23	0.42	0.110	0.231^*^	0.189	0.267^**^	−0.170	0.274^**^	(0.58)				
8	Conscientiousness	3.95	0.44	0.385^**^	0.244^*^	0.333^**^	0.317^**^	−0.082	0.199^*^	0.129	(0.72)			
9	Openness to experience	3.72	0.45	0.162	0.88	0.131	−0.007	−0.139	0.240^*^	0.040	0.178	(0.72)		
10	Role ambiguity	2.05	0.75	−0.499^**^	−0.660^**^	−0.632^**^	−0.063	0.166	−0.372^**^	−0.220^*^	−0.288^**^	−0.129	(0.82)	
11	Relational competence	4.04	0.51	0.404^**^	0.387^**^	0.426^**^	0.147	0.076	0.313^**^	0.141	0.360^**^	0.282^**^	−0.319^**^	(0.83)

*N* = 105; Teaching efficacy, School connectedness, Teacher subjective wellbeing = Criterion variables; Honesty-humility, Emotionality, Extraversion, Agreeableness, Conscientiousness, Openness to experience = Personality traits; Values of the internal consistency alphas are displayed in the diagonal; ^*^p < 0.05; ^**^p < 0.01.

*Socio-demographic variables* such as gender and type of higher education institution (civilian university or military academy) were also collected.

### 8.4. Data analysis

The statistical software package SPSS 27.0. was used for data analysis. To test the hypotheses, three hierarchical multiple regression analyses were conducted separately for *teaching efficacy*, *school connectedness,* and *teacher wellbeing* as criterion variables, and *personality traits*, *role ambiguity,* and *relational competence* as predictor variables. Personality traits were introduced in the first step because they are the most stable variables. Role ambiguity was introduced in the second step as it was considered to have a strong influence on teachers’ wellbeing, and relational competence was introduced in the last step, as a strong mediator between wellbeing and stress caused by the role ambiguity.

## 9. Results

The descriptive statistics and the correlation matrix between the research variables are included in Table 1.

The first hypothesis of this research stated that *personality traits positively relate to teaching efficacy, school connectedness, and teacher subjective wellbeing* and was partially supported by the results. *Emotionality* correlated negatively and significantly with teaching efficacy (*r* = −0.23, *p* = 0.015), school connectedness (*r* = −0.34, *p* < 0.001), and teacher subjective wellbeing (*r* = −0.31, *p* = 0.001). *Extraversion* correlated positively and significantly with teaching efficacy (*r* = 0.46, *p* < 0.001), school connectedness (*r* = 0.51, *p* < 0.001), and teacher subjective wellbeing (*r* = 0.53, *p* < 0.001). *Conscientiousness* also correlated positively and significantly with teaching efficacy (*r* = 0.38, *p* < 0.001), school connectedness (*r* = 0.24, *p* = 0.012), and teacher subjective wellbeing (*r* = 0.33, *p* = 0.001). *Agreeableness* correlated positively and significantly only with school connectedness (*r* = 0.23, *p* = 0,018). *Honesty-humility* and *openness to experience* did not correlate significantly with any of these three variables. In conclusion, only three of the six personality traits (emotionality, extraversion, and conscientiousness) correlate significantly with teaching efficacy, school connectedness, and teacher subjective wellbeing, while two personality traits (honesty-humility and openness to experience) do not correlate at all with these variables, and one of them (agreeableness) significantly correlates only with school connectedness.

The second hypothesis stated that *role ambiguity negatively relates to teaching efficacy, school connectedness, and teacher subjective wellbeing* and is fully supported by the results: *role ambiguity* correlated negatively and significantly with teaching efficacy (*r* = −0.49, *p* < 0.001), school connectedness (*r* = −0.66, *p* < 0.001), and teacher subjective wellbeing (*r* = −0.63, *p* < 0.001). Therefore, role ambiguity negatively correlates to teaching efficacy, school connectedness, and teacher subjective wellbeing.

The third hypothesis was formulated as follows: *teacher relational competence positively relates to teaching efficacy, school connectedness, and teacher subjective wellbeing, after controlling the effect of personality traits and role ambiguity.* Table 2 presents the results of the regression analysis with the three dependent variables.

**Table 2:** Hierarchical regression analysis predicting teacher efficacy, teacher school connectedness, and teacher subjective wellbeing.

Variables	Teaching efficacy			School connectedness			Teacher subjective wellbeing		
	*R* ^2^	Δ*R*^2^	*B*	*R* ^2^	Δ*R*^2^	*B*	*R* ^2^	Δ*R*^2^	*B*
**Step 2**	0.306^**^	0.306^**^		0.316^**^	0.316^**^		0.344^**^	0.344^**^	
Honesty-humility			0.034			−0.063			−0.019
Emotionality			−0.040			−0.132			−0.097
Extraversion			0.395^**^			0.414^**^			0.437^**^
Agreeableness			−0.053			0.092			0.028
Conscientiousness			0.297^**^			0.170			0.246^**^
Openness to experience			0.011			−0.064			−0.032
									
**Step 2**	0.396^**^	0.090^**^		0.543^**^	0.227^**^		0.526^**^	0.182^**^	
Honesty-humility			0.048			−0.040			0.001
Emotionality			−0.044			−0.137			−0.102
Extraversion			0.297^**^			0.258^**^			0.297^**^
Agreeableness			−0.094			0.027			−0.031
Conscientiousness			0.222^*^			0.050			0.139
Openness to experience			0.006			−0.072			−0.039
Role ambiguity			−0.335^**^			−0.533^**^			−0.476^**^
									
**Step 2**	0.421^*^	0.025^*^		0.576^**^	0.033^**^		0.560^**^	0.034^**^	
Honesty-humility			0.038			−0.053			−0.012
Emotionality			−0.097			−0.199^*^			−0.164^*^
Extraversion			0.243^*^			0.196^*^			0.234^**^
Agreeableness			−0.099			0.021			−0.036
Conscientiousness			0.179^*^			0.001			0.089
Openness to experience			−0.031			−0.114			−0.082
Role ambiguity			−0.304^**^			−0.497^**^			−0.440^**^
Relational competence			0.192^*^			0.218^**^			0.222^**^

*N* = 105; Teaching efficacy, School connectedness, Teacher subjective wellbeing = Criterion variables; Honesty-humility, Emotionality, Extraversion, Agreeableness, Conscientiousness, Openness to experience = Personality traits; ^*^p < 0.05; ^**^p < 0.01.

Regarding *teaching efficacy* as a criterion variable, in the first step, personality traits accounted for 30% of the variance and the model was significant [*F*(6,98) = 7.20, *p* < 0.001], with extraversion (*β* = 0.39, *p* < 0.001) and conscientiousness (*β* = 0.29, *p* = 0.002) as significant predictors. By adding the role ambiguity as independent variable in the second step of the regression model, and by controlling the influence of personality traits, the predictive value of the second model increases to 39% [Δ*R*^2^ = 0.090; *F*(1,97) = 14.42, *p* < 0.001], with role ambiguity as a significant predictor (*β* = −0.33, *p* < 0.001). In the third step, the relational competence was added to the regression model (*β* = 0.19, *p* = 0.043) resulting in an increase of the predictive value of the model to 42% [Δ*R*^2^ = 0.025, *p* = 0.043; *F*(1,96) = 4.21, *p* = 0.043], after controlling the influence of the personality traits and role ambiguity.

In what regards *school connectedness* as a criterion variable, in the first step, personality traits accounted for 31% of the variance and the model was significant [*F*(6,98) = 7.53, *p* < 0.001], with only extraversion (*β* = 0.41, *p* < 0.001) as a significant predictor. By adding in the second step of the regression model the role ambiguity as independent variable, and by controlling the influence of personality traits, the predictive value of the second model increases to 54% [Δ*R*^2^ = 0.22; *F*(1,97) = 48.27, *p* < 0.001], with role ambiguity as significant predictor (*β* = −0.53, *p* < 0.001). In the third step, relational competence was added to the regression model (*β* = 0.21, *p* = 0.007), resulting in an increase of the predictive value of the model to 57% [Δ*R*^2^ = 0.033, *p* = 0.007; *F*(1,96) = 7.47, *p* = 0.007], after controlling the influence of the personality traits and role ambiguity. In the third step of the regression, emotionality becomes a significant predictor for school connectedness (*β* = −0.19, *p* = 0.013).

Regarding *teacher subjective wellbeing* as a criterion variable, in the first step, personality traits accounted for 34% of the variance, and the model was significant [*F*(6,98) = 8.56, *p* < 0.001], with extraversion (*β* = 0.43, *p* < 0.001) and conscientiousness (*β* = 0.24, *p* = 0.007) as significant predictors. By adding the role ambiguity as independent variable in the second step of the regression model and by controlling the influence of personality traits, the predictive value of the second model increases to 52% [Δ*R*^2^ = 0.18; *F*(1,97) = 37.20, *p* < 0.001], with role ambiguity as a significant predictor (*β* = −0.47, *p* < 0.001). In the third step, teacher relational competence was added to the regression model (*β* = 0.22, *p* = 0.008) resulting in an increase of the predictive value of the model to 56% [Δ*R*^2^ = 0.034, *p* = 0.008; *F*(1,96) = 7.44, *p* = 0.008], after controlling the influence of the personality traits and role ambiguity. In the third step of the regression, emotionality becomes a significant predictor for school connectedness (*β* = −0.16, *p* = 0.042).

These results indicated that teacher relational competence positively relates to teaching efficacy, school connectedness, and teacher subjective wellbeing, after controlling the effect of personality traits and role ambiguity. Therefore, the third hypothesis received full statistical support.

## 10. Discussion

This study aimed to examine the relationship between personality traits, role ambiguity, relational competence, and teacher subjective wellbeing and to identify the degree to which the first three variables predict the last, by using a sample of university teachers.

The first hypothesis was partially supported by the results: three of the six personality traits—emotionality, extraversion, and conscientiousness—significantly correlated with teaching efficacy, teacher school connectedness, and teacher subjective wellbeing, while one of the personality traits—agreeableness—significantly correlated only with school connectedness. Honesty-humility and openness to experience did not correlate significantly with any of these three variables. However, when these personality traits were introduced into the regression analysis, we observed that extraversion and conscientiousness remained predictors for teaching efficacy during each step of the analysis and only in the first step for teacher wellbeing; in steps two and three, conscientiousness loses its quality as a predictor for school connectedness and teacher wellbeing, being replaced by emotionality.

Extraversion remains the only personality trait that positively predicts all three dimensions of teacher wellbeing at work during all three steps of the regression analysis for the sample of university teachers included in this research. Highly extraverted individuals have a positive view of themselves, are confident in their abilities when leading or addressing groups of people, enjoy social interactions and encounters, and generally experience positive feelings of enthusiasm, energy, and optimism, feel popular, and enjoy being in the spotlight (Ashton and Lee, 2009). Consequently, the higher the level of the teachers’ extraversion, the stronger their sense of success in teaching and the more they value their professional achievements. These teachers love to interact with student groups both formally, in lectures and seminars, and informally, in extracurricular activities and meetings. They are enthusiastic and have a high level of energy and optimism which they project into their activities and relationships with students and peers.

The results obtained in this study are similar to those of other authors. Extraversion is the personality trait most strongly associated with subjective wellbeing (Costa and McCrae, 1980; Lee et al., 2008; Aghababaei and Arji, 2014). Extraversion and conscientiousness were also predictors of subjective wellbeing for teachers in the study conducted by Albuquerque et al. (2012). Positive affect, as a component of wellbeing, has a strong association with extraversion (Diener et al., 2003).

Conscientiousness predicts the teaching efficacy and the subjective wellbeing of the university teachers in the sample. Individuals with high scores on this trait consistently organize both their time and their physical environment, work in a disciplined manner to achieve their goals, strive to accurately solve tasks, do not shy away from difficult or challenging tasks, and deliberate carefully when making decisions (Ashton and Lee, 2009). Therefore, these characteristics participate in creating the sense of teaching effectiveness and subjective wellbeing at work.

Introducing role ambiguity as a predictor variable in the second step of the regression analysis excludes conscientiousness as a predictor for school connectedness and teacher wellbeing, but not for teaching efficacy. Therefore, this personality trait remains significant for the respondents’ sense of teaching efficacy, but not for their overall sense of wellbeing at work or their sense of connectedness to their higher education institution. Conscientiousness as a personality trait loses its ability to mitigate the stressful effects of role ambiguity; when teachers do not have enough information to successfully deal with their current tasks or cannot anticipate what tasks will be assigned to them in the future, they must turn to coping strategies in which they mobilize their cognitive and behavioral resources in order to adapt to stressful circumstances (Răducu and Stănculescu, 2022).

Furthermore, where teachers are affected by role ambiguity, conscientiousness becomes insignificant to their sense of connection to the university in which they work. In this case, teaching identification appears to be a factor with a strong mediating impact between role stress (role conflict and role ambiguity) and psychological distress: “Low levels of role ambiguity and role conflict were associated with high levels of teaching identification, which in turn was associated with low levels of anxiety and hopelessness […] Teachers with a strong professional identity may see their role as a calling rather than a job. Being a teacher is a matter of pride that generates a positive self-concept” (Pretorius and Padmanabhanunni, 2022, p. 9).

As stated in the second hypothesis, role ambiguity negatively related to teaching efficacy, school connectedness, and teacher subjective wellbeing. Uncertainty about job tasks, goals, and responsibilities at work induces a state of stress that may manifest itself in negative affective states, such as anxiety and depression. In the case of a university teacher, for example, the teaching and research tasks formally included in the job description may be supplemented by informal expectations that are not adequately defined or for which they may not be adequately prepared. The period of the coronavirus pandemic triggered significant changes in teachers work, with direct effects on their mental health. The new conditions imposed online teaching, hybrid teaching, asynchronous classes, and social distancing classes that added a consistent additional stress to this professional group that, even before the pandemic, had been considered as one of the most exposed to stress-related disorders (Răducu and Stănculescu, 2022). Teachers at all levels of education have had to restructure their teaching activities, learn how to use new technologies, and at the same time provide support to their students and parents on how to use them (Rad et al., 2022). The overlap of work and personal space and work and family responsibilities caused by remote online teaching has increased the psychological pressure felt by teachers; this has been supplemented by feelings of social isolation and limited contact with supportive resources, such as managers, peers, and school administrators (Baker et al., 2021). The effects were strong on their feelings of effectiveness in teaching and assessment, feelings of connectedness to the school, and general wellbeing.

Lastly, the research results confirmed the third hypothesis, which argued that teacher relational competence positively relates to teaching efficacy, school connectedness, and teacher subjective wellbeing, after controlling the effect of personality traits and role ambiguity.

The introduction of the relational competence as a predictor variable during the third step of the regression analysis highlights the importance of emotionality as a predictor of school connectedness and teacher subjective wellbeing. The HEXACO-60 model describes individuals with high emotionality scores as anxious, sensitive to low levels of stress, with a strong fear of physical danger, and with high emotional needs generating intense emotional attachments to other people. In our study, the higher the level of teachers’ emotionality, the lower their sense of connection to their university, their subjective sense of wellbeing, and teaching efficacy. Although relational competence adds a small percentage to the proposed model’s ability to predict teacher subjective wellbeing, we believe it may mitigate the negative effects of emotionality as a personality trait and role ambiguity as a professional stressor.

## 11. Theoretical and practical implications

Numerous studies indicate that personality is the strongest predictor of subjective wellbeing, with extraversion being associated with the individuals’ positive affects and neuroticism being associated with negative affects. Studies have indicated that the HEXACO model shows strong significant relationships with wellbeing constructs and its use in the study of these constructs is more advantageous than the use of the Big-Five or BIS-BAS models. The honesty-humility trait, for example, measured only by the HEXACO, appears not to be beneficial for a person’s subjective state of wellbeing (Aghababaei and Arji, 2014; Anglim et al., 2020).

This study focused on the power of the six-dimensional framework of personality structure of HEXACO to predict subjective wellbeing of university teachers. Our results are in line with the findings of other researchers that have used HEXACO and found extraversion to be the strongest predictor of wellbeing, followed by conscientiousness and emotionality.

With regard to role ambiguity, two main types of solutions are discussed in the literature: solutions that depend on the occupant of the role and solutions that depend on the management of the organization (Rogelberg, 2007).

Individually, teachers can engage in a process of self-reflection in which they identify the sources of their role ambiguity and their intrinsic bias in how they perceive this ambiguity. By using self-reflection methods and tools, teachers can directly self-assess their own skillset, interests, and level of adaptation to the current role and indirectly estimate their level of productivity, job satisfaction, and wellbeing, both in the current role and in possible future roles. On the other hand, universities can collect more information about how teachers perceive their roles. Based on this, they can identify the area of training needed to reduce role ambiguity, while simultaneously reducing the costs associated with teachers wellbeing and productivity problems. Employers can also intervene in job descriptions, formulating them so that they are much clearer in terms of the skills, competences, and responsibilities required by the job holder. Another proposed solution concerns the creation by universities of tools that can be included in workplace design: social media platforms, online engagement forums, “or even email profile description spaces, where they can regularly update their self-explained expertise and role descriptions, along with manager or peer-appraised testimonials” (Saha et al., 2019, p.137).

It is important to remember that, for some employees, role ambiguity can trigger proactive behaviors whereby they shape their role in a creative or innovative way. If this is the case for universities, they should recognize and reward those teachers who redefine the boundaries of their role, as they bring role and skill diversity to their profession and use a set of coping strategies (Samfira and Paloș, 2021) that could be extended across work communities (Wang et al., 2011; Saha et al., 2019).

The teachers’ relational competence can be improved through learning and practice, alongside teaching and classroom management competencies (Jensen et al., 2015,). “Thus, teachers need to know how to form, maintain, improve, and strengthen the quality of the relationships: how to work consciously and systematically with the relationship as a space for development and learning” (Vidmar and Kerman, 2016, p. 43). Relational competence is part of a broader category of social and emotional competencies (SEC), which can be grouped into five clusters: self-awareness, self-regulation, social awareness, relationship skills, and responsible decision-making (Schonert-Reichl et al., 2017). Teachers can follow different training programs to develop the competences they lack (Mara and Mara, 2011), but it is necessary to transfer these practices into their daily work. To facilitate this transfer, universities need to cultivate a type of culture that values the development of these skills and implement a set of policies that support their practical application (Jones and Bouffard, 2012).

Based on these results, modules (as component parts of courses), but also stand-alone courses, can be designed and included in the curriculum of initial and in-service teacher training programs, in order to contribute to increasing teachers’ performance. Therefore, in what follows, we will present some proposals, namely two possible training modules and one course, which can be included in teacher training and professional development programs offered by universities.

- A first proposal concerns a training module that can be generically called *Teacher Personality* (TP), focused on the analysis by teachers/future teachers of their own personality profile based on the Big-Five model, with emphasis on the analysis of the connection between each personality dimension, the teacher’s classroom behavior, and a series of concepts associated with job performance and teaching efficacy. During the TP module, learners can be introduced to and practice different teaching strategies and models of teaching behavior related to each personality dimension. Given that extraversion and conscientiousness appear to be the strongest predictors of teaching efficacy (Kim et al., 2019), effective teaching, and professional development strategies closely related to these dimensions can be practiced during the TP module classes. For example, a teacher with a low level of conscientiousness needs to learn and practice using effective ways of organizing and planning activities, time and competing priorities management, and general organization skills. On the other hand, introverted teachers need to learn and practice assertive communication, social interaction, and group management techniques. Highly emotional teachers need to learn emotional control techniques and low agreeableness teachers need to practice their ability to empathize and manage frustration. Although studies do not indicate a significant correlation of openness to experience with teaching efficacy, this dimension is an essential component of the continuing professional development that also helps promote the scientific research skills of university teachers. Therefore, it needs to be developed through methods that stimulate motivation for lifelong learning.
- The second proposal concerns a course that could be called *Professional Stress Management* (PSM) and that could be included in the offer of universities for continuous teacher training. The PSM course should incorporate information on strategies for managing role stress, focusing on identifying stressors, and managing personal reactions to them, on building healthy coping strategies, setting personal boundaries, getting support from supervisors and colleagues, and accessing the resources needed to perform the role effectively. Role stress consists of role conflict (generated by incompatible expectations that hinder role performance), role overload (role expectations that exceed the employee’s resources), and role ambiguity (unclear role expectations that fail to guide the employee’s behaviors; Hindin, 2007). The practical-applicative value of this course can be enhanced by conducting case studies and group discussions using both the theoretical knowledge acquired and the personal experience of the trainee-teachers.
- A third proposal concerns a training module that can be generically called *Teacher Relational Competence* (TRC), built around the “relational competence model” developed by Aspelin et al. (2021). In the TRC module, trainee-teachers can be challenged to analyze problematic educational sequences, as well as also typical cases. Role-plays related to the management of teacher-student relationships can be organized, aimed at developing the three dimensions of teacher relational competence, as follows:

- *Communicative competence*, based on those skills that enable the teacher to harmonize with students during verbal and non-verbal communication;
- *Differentiation competence,* focusing on the skills that help the teacher regulate physical and psychological distance in their relations with the students;
- *Socio-emotional competence,* pivoting on those skills that help teachers manage the emotional indicators that arise as part of their relationships with the students, including their own emotions as well as the students.

## 12. Limitations and future directions

The first limitation of this study is generated by the sample size and structure. This sample is not representative of the universities from which the respondents come, much less of the body of university teachers in Romania, neither by size nor by structure. Being a convenience sample, the results of the study cannot be generalized. Self-report measures were used as research instruments, which are generally affected by social desirability bias. As it is a cross-sectional study, its results represent a picture of teacher subjective wellbeing limited to a certain point in time. In addition, while personality traits are relatively stable over time, wellbeing and role ambiguity may vary by time and situation. However, the results of the study are consistent with those obtained by similar studies.

The second limitation of the study is that it does not allow the identification of causal relationships between variables. Further studies could identify, for example, the type of relationship between personality traits and wellbeing, teachers’ relational competence, and their perception of role ambiguity/clarity or, moreover, role stress, using other methods such as observation or experiment. Longitudinal studies could identify, for example, the stability over time of the links between the variables studied.

## 13. Conclusion

Investigating teacher wellbeing and understanding the relationships between it and a range of psychological or social factors is an important scientific endeavor as, beyond the effects on individuals themselves, teacher wellbeing also impacts students and can therefore have important long-term effects (Renshaw et al., 2015a). Without the teachers’ wellbeing, it is hard to build up students’ wellbeing (Konu et al., 2010). “Cultivating teacher wellbeing it is necessary not just because doing so makes them feel good and satisfied, but also because doing this will transform them to be more engaged, with better results in their activities” (Stănculescu, 2014, p.38).

By examining the relationship between teacher subjective wellbeing, personality, role ambiguity, and relational competence, this paper extended the results of previous studies on teachers’ work-related wellbeing. In this research, university teachers’ personality traits (extraversion, conscientiousness, and emotionality), role ambiguity, and relational competence predicted their teaching efficacy, school connectedness, and subjective wellbeing. The theoretical and practical implications of these results may suggest a series of contents for teacher training programs or for university policies aimed at improving the quality of work and professional life of their employees.

## Data availability statement

The raw data supporting the conclusions of this article will be made available by the authors, without undue reservation.

## Ethics statement

Ethical review and approval was not required for the study on human participants in accordance with the local legislation and institutional requirements. Written informed consent for participation was not required for this study in accordance with the national legislation and the institutional requirements.

## Author contributions

CM: conceptualization, data collection and processing, and writing—original draft, reviewing, and editing. SB: conceptualization, data collection, and writing—original draft, reviewing, and editing. FM-B: data collection and writing—reviewing. All authors contributed to the article and approved the submitted version.

## Conflict of interest

The authors declare that the research was conducted in the absence of any commercial or financial relationships that could be construed as a potential conflict of interest.

## Publisher’s note

All claims expressed in this article are solely those of the authors and do not necessarily represent those of their affiliated organizations, or those of the publisher, the editors and the reviewers. Any product that may be evaluated in this article, or claim that may be made by its manufacturer, is not guaranteed or endorsed by the publisher.
